# Sheltering the People

**Published:** 1923-05

**Authors:** 


THE
### HOSPITAL and HEALTH REVIEW

### SHELTERING THE PEOPLE.

NOTWITHSTANDING that the provisions of the new Government Housing Bill were already fairly well known in outline before it was printed it is impossible to repress a feeling of disappointment now that its details are available. It can only be looked at as an instalment of the country's needs. In the nature of things it cannot supply more than a fraction of those needs, since as it stands it seeks mainly to provide dwellings of the smallest type. Mr. Neville Chamberlain has talked of limiting Government assistance to houses of the "non-parlour" variety, but the measure itself contains no reference to that variety—it is merely implied in the limitations of superficial feet which it lays down. In themselves the reasons for the limitation of size are good so far as they go, but it will be for Parliament and the country to say whether it considers them to be sufficient. The smallest class of artisans' dwelling, whether self-contained cottages or flats, does not attract private capital for the conclusive reason that such buildings are too costly to allow of anything approaching an economic return. It is estimated, indeed, that the annual loss on each subsidised dwelling will average £12, one-half of which will be found by the Exchequer. It is not unlikely that the loss will sometimes be greater, but in no case is the subvention from public funds to exceed £6 a year for twenty years. The local authority is to be the immediate executive power, and it is given certain options as to the form in which its share of the encouragement to building shall take. It may grant a lump sum for each house, relieve it from rates for a specified period, provide the interest due to building societies, or repay advances made by them. These alternatives, however, are still limited to houses of the specified dimensions. Further, the municipality may advance money to private builders directly in cash, or may guarantee advances from building societies. This is a distinctly valuable provision, but it is essential that it should be made thoroughly well known, and that the local authorities shall be urged to press it upon their constituencies. It embraces houses up to a value of £1,500—a limit of value which should assist materially in the provision of those larger dwellings which are almost as badly needed as the "non-parlour" type.

Another useful provision is that which places public utility societies in the same position as the local authorities in regard to the subvention, while the valuable, but hitherto almost inoperative, Small Dwellings Acquisition Act is extended from houses costing £800 to those costing £1,200. This, however, is less an extension of facilities than a recognition of the lowered value of money. These various alternatives give the Bill a degree of elasticity which, although it is smaller than could have been wished, widen its scope sufficiently to give it a fair chance of the modified success which alone is possible for such a measure. As we have said, it is an instalment, and it would be unfair and absurd to criticise it as though it were put forward as a panacea. No one, we are sure, realises its restrictions more acutely than its author. Mr. Neville Chamberlain knows, from experience, the difficulties of the housing question at any time, to say nothing of the present moment, more thoroughly than any of his predecessors at the Ministry of Health. He is doing what he can, rather than what he would, and the country is in the same position. That is not to say that the Bill is incapable of improvement. It can be, and probably will be, improved before it receives the Royal Assent. It is exceedingly desirable, for example, that it should aid the erection of a rather better type of house, even if we have to find another pound, or two pounds, a year to enable such houses to be built. To put up, perhaps, a hundred thousand tiny tenaments with no bathrooms or other amenities and with rigidly limited space would be a retrograde step that could in no way conduce to the comfort, self-respect, or health of their occupants. Let us not forget that this measure emanates from a Ministry of Health, and that if the nation's money is to be spent upon housing it must be expended upon dwellings which will not be the despair of our children.

We readily recognise that the Bill is an honest attempt to deal with an acute difficulty, but it only touches the fringe of that difficulty and there is plenty of room for improvement in directions which space fails us to enumerate. At the same time we are satisfied that some of its critics are talking sheer nonsense, or are actuated by motives which have small concern with the merits of the subject, and take little account of the true interests of those whom it seeks to benefit. Thus the Standing Joint Committee of Industrial Women's Organisations describes the Bill as a " Charter to the jerry-builder." That is mere hysterical rubbish. There are such things as building by-laws and Council surveyors, both of them distinctly formidable facts, which make jerry-building an exceedingly difficult enterprise. The official attitude of the Labour Party is even more obscurantist. It is the announced intention of that party to propose the rejection of the Bill, which is about the most futile thing it could do. It would be far better advised to bring its experience of the needs of the working classes to the improvement of the measure. It might also show a little mercy towards that small *bourgeoisie* whose needs are nearly, if not fully, as urgent as its own. All men who wear black coats are not "Capitalists," and the straits to which the lower ranks of the salaried and fixed-income classes have for years past been put in the matter of house-room have been exasperating. Our worst fear in regard to the Bill is that it will fall very far short of the country's needs and that, a year or two hence—the subsidy is available only to September 30, 1925—we shall still be facing a serious scarcity of houses. It is almost inevitable that it should be so. It is to private capital alone that we can look in the future, as we have looked in the past, for a sufficiency of dwellings of all sizes and for all classes, and so long as there is no possibility of a reasonable return for the use of that capital we shall look towards it in vain. Private enterprise was killed for the time being by the restrictions which are about to be renewed for a further, if limited, period, and until free trade in housing is restored, private enterprise will hardly move. That is the central fact of the situation. Once we have shown the builder and those who finance him that their fears that control will be permanent have no foundation, more houses will be built than will ever be forthcoming as the result of a system of mixed State and municipal subsidies.

